# C-Reactive Protein: Higher During Acute Psychotic Episodes and Related to Cortical Thickness in Schizophrenia and Healthy Controls

**DOI:** 10.3389/fimmu.2018.02230

**Published:** 2018-10-10

**Authors:** Isabella Jacomb, Clive Stanton, Rohini Vasudevan, Hugh Powell, Maryanne O'Donnell, Rhoshel Lenroot, Jason Bruggemann, Ryan Balzan, Cherrie Galletly, Dennis Liu, Cynthia S. Weickert, Thomas W. Weickert

**Affiliations:** ^1^Schizophrenia Research Laboratory, Neuroscience Research Australia, Randwick, NSW, Australia; ^2^Prince of Wales Hospital, Randwick, NSW, Australia; ^3^School of Psychiatry, University of New South Wales, Randwick, NSW, Australia; ^4^Discipline of Psychiatry, University of Adelaide, Adelaide, SA, Australia; ^5^College of Education, Psychology and Social Work, Flinders University, Adelaide, SA, Australia; ^6^Northern Adelaide Local Health Network, Adelaide, SA, Australia; ^7^Department of Neuroscience and Physiology, Upstate Medical University, Syracuse, NY, United States

**Keywords:** schizophrenia, acute psychosis, c-reactive protein, cortical thickness, inflammation, working memory, cognition, CRP

## Abstract

There is increasing evidence for the role of inflammation in schizophrenia, yet the stability of increased peripheral inflammation in acute psychosis and the degree to which peripheral inflammation relates to cortical thickness, a measure of the degree of neuropathology, are unknown. In independent samples, we assessed the peripheral inflammation marker C-reactive protein (CRP) to determine the extent to which: (1) CRP was elevated and stable across admissions for acute psychosis, (2) cognition, daily function and symptom severity are characteristic of chronically ill patients with schizophrenia displaying elevated CRP, and (3) CRP levels predict cortical thickness. Study 1 assessed peripheral CRP (primary outcome) and other blood measures in 174/280 people with acute psychosis while Study 2 assessed peripheral CRP, cognition and cortical thickness (primary outcomes), symptoms, and daily function in 85/97 chronically ill patients with schizophrenia and 71/87 healthy controls. In acute psychosis, CRP and neutrophil-to-lymphocyte ratio were significantly elevated relative to a normal cutoff (with 59.8% of patients having elevated CRP) which remained elevated across admissions. CRP was significantly elevated in 43% of chronically ill patients with schizophrenia compared to 20% in controls. Elevated CRP patients displayed significantly worse working memory and CRP was inversely correlated with cortical thickness in frontal, insula, and temporal brain regions. This work supports the role of inflammation in psychotic illnesses and suggests that use of peripheral markers (e.g., CRP) in conjunction with diagnosis could be used to identify patients with more cortical neuropathology and cognitive deficits.

## Introduction

Schizophrenia is a severe psychiatric disorder characterized by positive (e.g., hallucinations and delusions) and negative (e.g., lack of motivation) symptoms, cognitive deficits, and functional decline. Presently there is no inexpensive, easily accessible diagnostic biomarker for the degree of cortical pathology in schizophrenia and substantial heterogeneity is associated with the disorder (1). While there are likely to be multiple causative pathways for developing schizophrenia (2), there is accumulating evidence to support the role of inflammation as increasing risk at least for a subset of patients (3). Therefore, improving interventions for schizophrenia may depend on identifying subgroups of patients characterized by inflammatory markers that can be targeted with specific treatments.

Recently, there has been increasing evidence that immunological and inflammatory mechanisms may play important roles in the pathophysiology of schizophrenia and related psychotic disorders (4–6). Epidemiological and preclinical evidence implicates prenatal infection and subsequent immune activation in the etiology of schizophrenia (7). Autoimmune disorders are significantly more prevalent in patients with schizophrenia compared to the general population (8, 9). It has been shown that antipsychotics, the most common treatment for psychosis, can decrease circulating levels of inflammatory markers which may, in turn, reduce psychotic symptoms (10, 11). However, some studies show an increase in peripheral inflammatory markers with second generation antipsychotics (12). Results from clinical trials of anti-inflammatory agents such as aspirin (13), non-steroidal anti-inflammatory drugs (14), and the microglial inhibitor minocycline (15, 16) suggest that adjunctive anti-inflammatory treatments may be a promising therapeutic approach for reducing symptoms and reversing cognitive deficits in people with schizophrenia.

Various immune and inflammatory alterations in the brain and blood have been found in subgroups of people with schizophrenia, including increased levels of pro-inflammatory cytokines (17–19), changes in white blood cell count (WBC) (20), a higher prevalence of positive antinuclear antibodies (ANA) (21, 22), increased neutrophil-to-lymphocyte ratio (NLR) (23, 24), and elevated C-reactive protein (CRP) (25, 26). Increases in inflammatory markers have been shown in subsets of individuals in both the prodromal stage (27, 28) and first-episode psychosis (29), suggesting that inflammation is not secondary to chronic illness or initiation of antipsychotics. The proportion of chronically ill patients with schizophrenia showing increased inflammation is generally quite substantial with ~40% who display elevated cytokine mRNA in the prefrontal cortex (17) and ~40% who display elevated cytokine mRNA in peripheral blood (30). These findings are consistent with meta-analytical results showing that cytokines are increased in the serum of people with schizophrenia (31). Furthermore, elevated proinflammatory cytokines, e.g., IL-6 and IL-1β, are inversely related to cognition and frontal brain volume in chronically ill people with schizophrenia (30). Thus, elevated inflammation markers appear to be a characteristic of psychosis in a substantial proportion of patients and they are related to brain structure and function.

CRP is an acute-phase reactant protein mainly produced by hepatocytes in response to an increase in circulating pro-inflammatory cytokines (32). CRP is attractive for use as a biomarker in psychiatry as it appears to be fairly stable, is easily and consistently measured by most diagnostic labs, and is considered a reliable biomarker of systemic inflammation (33–35). The healthy normal clinical range of CRP levels is generally < 3 mg/L (36), whilst CRP > 2 mg/L has been associated with increased risk of cardiovascular disease (37). Recent meta-analyses have reported a high prevalence of elevated CRP in schizophrenia (25, 36) and an association between adolescent CRP and adult schizophrenia diagnosis has been found in a recent longitudinal study (38). Elevated CRP has been associated with impaired cognitive functioning in chronically ill patients with schizophrenia (39, 40) and with acute psychotic episodes (41). An association between elevated CRP and more severe psychiatric symptoms in schizophrenia has also been shown (42); however, other studies have failed to replicate this finding (40, 43). Other studies suggest that CRP may be particularly elevated during exacerbations of psychosis (44) and may decrease following resolution of an acute psychotic episode (45). Whilst a small number of studies have shown an association between elevated CRP and decreased structural brain volume in healthy older adults (46, 47), there have been no studies to date assessing the potential link between CRP and cortical thickness in schizophrenia.

Here, we assess CRP as a marker of inflammation in two independent samples of patients with psychosis. The aims of Study 1 were to determine the extent to which: (1) CRP levels were elevated in a proportion of patients presenting with an acute psychotic episode, (2) relationships exist between CRP and other inflammation markers (including NLR, WBC, and ANA), and (3) peripheral inflammation markers remain stable over repeated admissions for acute psychotic episodes. We made the following three hypotheses: (1) CRP would be elevated above 3 mg/L in a subgroup of patients with acute psychosis, (2) there would be a significant difference in other peripheral inflammation markers between patients grouped on the basis of elevated and normal CRP, and (3) CRP would be consistently elevated during subsequent admissions for acute psychotic episodes in the same patients.

Given the limitations of assessing acutely psychotic patients for brain and cognitive measures, in Study 2 we tested the extent to which a peripheral inflammation marker (CRP) was related to cognition and a brain measure (cortical thickness) in a cohort of chronically ill patients with schizophrenia who were not acutely psychotic. The aims of Study 2 comparing chronically ill patients with schizophrenia to a healthy control group were to determine the extent to which: (1) CRP levels were elevated, (2) the proportions of patients versus controls having elevated CRP, (3) there were significant cognitive, symptom severity, and daily function differences between patients with elevated and patients with normal CRP levels, and (4) CRP levels predict cortical thickness. We made the following four hypotheses: (1) CRP would be significantly elevated in a subgroup of chronically ill patients with schizophrenia and schizoaffective disorder relative to healthy controls, (2) cognition, symptom severity, and functional abilities would be significantly affected in patients with schizophrenia who display elevated CRP relative to those patients who display normal CRP levels, (3) CRP levels would negatively correlate with cortical thickness, and (4) CRP would be elevated to a lesser extent in chronically ill patients compared to acutely ill patients (comparing results from Study 1 and Study 2).

## Study 1: CRP in patients with an acute psychotic episode

### Materials and methods

#### Study design

##### Participants

Participants consisted of patients with an acute psychotic episode who were admitted to the Mental Health Intensive Care Unit (MHICU) at the Prince of Wales Hospital, Sydney, Australia, between March 2013 and February 2016. The MHICU is a 12-bed inpatient unit that provides treatment for patients who are acutely and severely mentally ill. A current comprehensive psychiatric assessment, current physical examination, and the last 7 days of progress notes were performed/documented prior to admission. The Prince of Wales Hospital MHICU admission criteria consisted of the following: (1) the patient is suffering with a mental disorder or illness which may have complex comorbidity, (2) the patient is presenting with behavioral difficulties, which seriously compromise the patient's physical wellbeing, psychological wellbeing, the physical wellbeing of others or the psychological wellbeing of others, (3) the patient's risk profile includes some or all of a significant risk of aggression, absconding with associated serious risk, suicide, or vulnerability, (4) it has been demonstrated that multidisciplinary management strategies have not succeeded in containing the presenting problems in the referring Mental Health Service, (5) the patient is detained under a section of the New South Wales Mental Health Act (2007), (6) the patient's risk profile is such that he/she does not require a higher level of security than that offered by the MHICU. The MHICU exclusion criteria (categories of patients who should not be treated on the MHICU) consisted of the following: (1) patients younger than 18 years, (2) patients older than 65 years, (3) patients who require a higher level of security by virtue of the risk profile, (4) patients with a primary diagnosis of substance misuse, where the current presenting behavior is a direct result of the substance misuse and not an exacerbation of a mental illness, (5) patients with a primary diagnosis of a personality disorder should not routinely be admitted to the MHICU, (6) patients with a primary diagnosis of dementia, (7) patients with a primary diagnosis of a learning disability, (8) patients with an exacerbated physical condition, which cannot be safely managed in the MHICU, (9) pregnant women, who would only be admitted to the MHICU in absolutely exceptional circumstances.

All patients were assessed and diagnosed using DSM-IV-TR criteria by trained psychiatrists. Individuals admitted to the MHICU without a psychotic disorder diagnosis were excluded. Patients who did not have CRP measured (*n* = 76; 27.1% of patients) were excluded. We also excluded patients suffering from any physical illness that may have influenced their immunological functioning (including non-Hodgkins lymphoma, Hashimoto's thyroiditis, HIV-AIDS and recent septicaemia; *n* = 5), and patients with inflammatory markers that were ≥ ± 2 standard deviations from the sample mean (*n* = 11).

##### Patients with single admissions

Out of a total of 280 individuals screened for inclusion, 106 individuals were excluded based on the above criteria, leaving a total of 174 individuals (117 males, 57 females) for entry into analyses. Patients were between the ages of 15 and 76 years old. Table 1 provides diagnostic frequencies of the acutely psychotic sample.

**Table 1:** Diagnostic classifications of the acute psychosis patient sample according to DSM-IV-TR.

	***n***	**Cumulative frequency**	**%**
Schizophrenia	60	60	34.48
Schizoaffective Disorder	45	105	25.86
Bipolar Disorder I and II	39	144	22.41
Psychotic Disorder NOS	13	157	7.47
Substance-Induced Psychotic Disorder	8	165	4.60
Brief Psychotic Disorder	2	167	1.15
Major Depressive Disorder (with psychotic features)	2	169	1.15
Manic Episode	2	171	1.15
Schizophreniform Disorder	2	173	1.16
Psychotic Disorder due to a general medical condition^†^	1	174	0.57

DSM-IV-TR, Diagnostic and Statistical Manual of Mental Disorders – Fourth Edition-TR;

† *Huntington's Disease*.

##### Patients with multiple admissions

Thirty patients with 2 admissions (23 males, 7 females), between 19 and 57 years of age (*M* = 34.8, *SD* = 10.3) at first admission, were included in the longitudinal analysis. Diagnoses included bipolar disorder (*n* = 10), schizoaffective disorder (*n* = 11), schizophrenia (*n* = 8), and psychosis NOS (*n* = 1). The mean length of time between admissions was 295.9 days (*SD* = 261.2; range = 13 to 898 days).

#### Blood collection and assays

Venous peripheral blood was collected within 1 week of admission. Whole blood samples were shipped on ice to the South-Eastern Area Laboratory Service where they were processed using a chemiluminessence assay. Laboratory blood tests routinely performed upon admission included: full blood count, CRP and ANA. WBC and NLR were obtained from full blood count results. Results of these blood tests were collected from electronic medical records and entered into a separate database for analysis. CRP results recorded as < 1 mg/L (*n* = 25) were entered in the database at the minimum detection level (0.3 mg/L).

#### Clinical data

Clinical data collected from patients included body mass index (BMI), smoking status (smoker/non-smoker), illicit substance use (drug-user/non-drug user; polysubstance abuse/non-polysubstance abuse) and antipsychotic treatment upon admission. See Table 2 for the frequency of patients receiving different antipsychotics. The Health of the Nation Outcome Scales (HoNOS), an indicator of health and social functioning, was administered to the majority of patients (48).

**Table 2:** Antipsychotic medication status at admission in acute psychosis patient sample.

	***n***
Olanzapine	49
Quetiapine	19
Zuclopenthixol decanoate/olanzapine	12
Chlorpromazine	9
Risperidone	8
Paliperidone depot/olanzapine	8
Aripiprazole	7
Amisulpride	4
Clozapine	4
Zuclopenthixol decanoate/chlorpromazine	4
Clozapine/olanzapine	3
Haloperidol	3
Paliperidone depot	3
Zuclopenthixol acetate	3
Risperidone depot/risperidone	2
Zuclopenthixol decanoate	2
Zuclopenthixol decanoate/quetiapine	2
Fluphenazine depot/chlorpromazine	2
Risperidone depot/olanzapine	2
Zuclopenthixol decanoate/risperidone	2
Haloperidol depot	1
Olanzapine depot/olanzapine	1
Zuclopenthixol acetate/olanzapine	1
Paliperidone depot/paliperidone	1
Zuclopenthixol acetate/quetiapine	1
Clozapine/aripiprazole	1
Haloperidol/quetiapine	1
Zuclopenthixol acetate/olanzapine	1
Amisulpride/olanzapine	1
Paliperidone depot/quetiapine	1
Zuclopenthixol acetate/chlorpromazine	1
Risperidone depot	1
Risperidone/quetiapine	1
Aripiprazole depot/aripiprazole	1
Flupenthixol depot	1
Risperidone/chlorpromazine	1
Aripiprazole depot/olanzapine	1
Flupenthixol depot/olanzapine	1
Clozapine/quetiapine	1
Paliperidone depot/chlorpromazine	1
Zuclopenthixol decanoate/zuclopenthixol	1

#### Statistical analyses

All data analyses were performed using SPSS version 23. Antipsychotic dose was converted to mean daily chlorpromazine (CPZ) equivalent dose based on standard guidelines (49). Effect size calculations were measured as Cohen's *d* (50).

For single admission analyses, a one-sample *t*-test was used to compare the average levels of CRP with a clinical cut-off of 3 mg/L (36). Participants were also grouped into two categories; “normal CRP” with CRP < 3 mg/L and “elevated CRP” with CRP ≥ 3 mg/L. Demographics, clinical variables and other inflammatory markers were compared between elevated and normal CRP groups using ANOVA or χ^2^-tests where appropriate. ANCOVA was used to compare NLR and WBC between CRP groups, covarying for age and BMI. For the multiple admissions analyses, repeated measures ANCOVAs (with age at initial admission as a covariate) were used to determine significant differences in mean daily CPZ equivalent dose, HoNOS, CRP, NLR, and WBC between initial and repeat admissions.

#### Study approval

This study was a retrospective analysis of patient's records blind to any personal identifying information. The study was conducted under protocol 15/028 approved by the South-Eastern Sydney Local Health District Human Research Ethics Committee.

### Results

#### Sample characteristics

Overall patient demographics, clinical, and inflammation measures are shown in Table 3. The numbers of patients classified by psychiatric diagnosis and the numbers of patients receiving different antipsychotic combinations are provided in Tables 1, 2. Mean CRP for the sample was 6.1 (*SD* = 6.7), which was significantly higher than the clinical cut-off of 3, *t*_(173)_ = 6.15, *p* < 0.01, *d* = 0.47. Mean NLR of 2.7 (*SD* = 1.1) was also significantly elevated relative to the clinical cut-off for NLR of 2.2, *t*_(170)_ = 5.88, *p* < 0.01, *d* = 0.45. Mean WBC was 7.8 (*SD* = 2.1), which was within the clinically normal range.

**Table 3:** Demographics, clinical and inflammation measures in the acute psychosis sample.

	***n***	**Mean (SD)**	**Normal range**
Age (years)	174	38.7 (13.6)	
**Sex**			
Male/Female (%)	117/57	67.2%/32.8%	
**Ethnicity**			
Caucasian/Other	109/65	62.6%/37.4%	
BMI	122	27.2 (6.4)	18.5–24.9
Smokers (%)	105	62.1%	
Drug users (%)	91	52.3%	
PSA (%)	56	32.2%	
CPZ equivalent dose	169	1185.9 (1628.2)	
HoNOS	134	15.5 (9.0)	
CRP	174	6.1 (6.7)	< 3.0
NLR	170	2.7 (1.1)	< 2.2
WBC	166	7.8 (2.1)	3.5–11
ANA positive (%)	42	66.7%	

*PSA, polysubstance abuse; CPZ, mean daily chlorpromazine; HoNOS, Health of the Nation Outcome Scale; BMI, body mass index; CRP, C-reactive protein; NLR, neutrophil-to-lymphocyte ratio; WBC, white blood cell count. Unless otherwise specified, values refer to means and s.d. in parentheses*.

The majority of people with acute psychosis (*n* = 104, 59.8%) had CRP levels ≥ 3 mg/L. Elevated NLR (≥ 2.2) was present in 70.2% (*n* = 73) of individuals with elevated CRP, but only 6.7% (*n* = 7) had elevated WBC (≥ 11). The majority of the people with acute psychosis who were tested for signs of autoimmunity had positive ANA titers (66.7%, i.e., 42/63 patients).

#### Subgroup analyses based on CRP levels

As shown in Table 4, those with acute psychosis and elevated CRP were significantly older and had a significantly greater BMI than individuals who were acutely psychotic but had normal CRP at the time of blood draw. There were no significant differences between the CRP subgroups (≥3 mg/L vs. < 3 mg/L) in relation to antipsychotic dosage, smoking status, drug use status, polysubstance abuse (PSA), HoNOS scores, or percent ANA positive. After co-varying for age and BMI, NLR remained significantly higher in patients with elevated CRP compared to patients with normal CRP. There was a trend toward a significant increase in WBC in the elevated CRP group relative to the normal CRP group after covarying for age and BMI.

**Table 4:** Characteristics of the acute psychosis sample in the normal (CRP < 3 mg/L) and elevated (CRP ≥ 3 mg/L) CRP groups.

	***n***	**“Normal” CRP < 3 mg/L**	***n***	**“Elevated” CRP ≥3 mg/L**	***F* or chi- square**	***p***	**Cohen's d**
Age	70	35.4 (12.8)	104	40.9 (13.6)	*F* = 7.26	0.008^*^	0.42
BMI	54	24.8 (5.4)	68	29.1 (6.6)	*F* = 15.00	< 0.001^*^	0.71
Smokers (%)	45	42.9%	60	57.1%	χ^2^ = 0.79	0.37	-
Drug users (%)	38	41.8%	53	58.2%	χ^2^ = 0.19	0.67	-
PSA (%)	25	44.6%	31	55.4%	χ^2^ = 0.67	0.51	-
CPZ equivalent	68	1067.9 (1589.6)	101	1265.4 (1656.8)	*F* = 0.60	0.44	0.12
HoNOS	56	15.3 (10.0)	78	15.6 (8.2)	*F* = 0.04	0.84	0.04
NLR^a^	53	2.4 (1.0)	66	2.9 (1.1)	*F* = 7.75	0.006^*^	0.48
WBC^a^	53	7.3 (2.0)	63	8.1 (1.9)	*F* = 3.22	0.08	0.41
ANA positive (%)	18	42.9%	24	57.1%	χ^2^ = 0.13	0.72	-

*CRP, C-reactive protein; BMI, body mass index; CPZ, chlorpromazine; PSA, polysubstance abuse; HoNOS, Health of the Nation Outcome Scale; NLR, neutrophil-to-lymphocyte ratio; WBC, white blood cell count; ANA, anti-nuclear antibodies. Unless otherwise specified, values refer to means and s.d. in parentheses*.

a *ANCOVA results covaring for age and BMI*.

* *p < 0.01*.

As shown in Table 5, CRP levels were significantly reduced at repeat admission compared to initial admission. However, mean CRP was significantly elevated relative to the clinical cut-off at both initial admission (*M* = 12.1, *SD* = 12.0), *t*_(29)_ = 4.18, *p* < 0.001, and repeat admission (*M* = 7.8, *SD* = 8.0), *t*_(29)_ = 3.29, *p* = 0.003. There were no significant differences in NLR and WBC between admissions. NLR was significantly elevated relative to the clinical cut-off at initial admission (*M* = 2.9, *SD* = 1.6), *t*_(27)_ = 2.35, *p* = 0.03, and repeat admission (*M* = 2.9, *SD* = 1.2), *t*_(27)_ = 3.02, *p* = 0.005. Mean WBC was within normal levels at both admissions. There were no significant differences in relation to mean daily CPZ equivalent dose and HONOS scores between admissions.

**Table 5:** Difference in inflammatory markers at initial and repeat admission with acute psychosis.

	***n***	**Initial admission**	**2^nd^ admission**	**Mean difference (SD)**	**F**	***p***	**Cohen's d**
CPZ equivalent	28	1700.2 (1877.8)	1797.9 (2095.4)	−97.7 (2135.1)	0.95	0.34	0.05
HONOS	18	14.7 (6.4)	12.7 (6.3)	−2.0 (6.8)	0.01	0.93	0.31
CRP	30	12.1 (12.0)	7.8 (8.0)	4.3 (16.3)	4.67	0.04^*^	0.42
NLR	28	2.9 (1.6)	2.9 (1.2)	0.1 (1.4)	2.89	0.10	0.00
WBC	27	8.3 (2.4)	8.8 (2.8)	−0.5 (3.6)	0.35	0.56	0.19

*Repeated measures ANCOVA covarying for age at initial admission. CRP, C-reactive protein; NLR, neutrophil-to-lymphocyte ratio; WBC, white blood cell count. Unless otherwise specified, values refer to means and s.d. in parentheses*.

* *p < 0.05*.

### Discussion

This study found CRP levels to be significantly elevated in patients with acute psychosis compared to clinical cut-offs, supporting previous work (45, 51). A large proportion (~60%) of the current sample had a CRP above the normal clinical range. NLR, which is the ratio of the number of most abundant white blood cells (neutrophils) to the total number of lymphocytes (which includes T cells, B cells. monocytes and Natural Killer Cells), was also significantly elevated in this acutely psychotic sample, confirming previous reports of increased NLR in schizophrenia (23, 24). WBC was within normal levels; however, there was a trend toward WBC being higher in the elevated CRP group compared to the normal CRP group, which is consistent with findings from a recent study that reported a positive relationship between leukocytes and CRP in 213 patients with schizophrenia (52). Furthermore, a large proportion of patients (~67%) in the current sample had positive ANA, which is a much higher frequency than previously reported (~20%) in chronically ill patients with schizophrenia (22) and higher than reported for healthy individuals (~26%) (53). Since known autoimmune disorders was an exclusion for being included in our analyses, elevated inflammatory markers would most likely not be attributed to other physical disorders in this instance.

We also found that there was a significant decrease in CRP levels at repeat admission compared to initial admission; however, CRP at both admissions was significantly elevated compared to the clinical cut-off. There was no significant change in NLR or WBC across both admissions. These findings demonstrate that CRP and NLR (but not WBC) are consistently elevated above normal levels during acute psychotic episodes. This may indicate that a subset of acutely psychotic individuals with elevated CRP or NLR may benefit most from adjunctive anti-inflammatory treatments initially at admission or later, possibly to prevent further admissions.

However, there are some potential confounding factors that make the connection between inflammation and psychotic illnesses uncertain. There was a significant difference between normal and elevated CRP groups in relation to age and BMI, which is consistent with previous findings that higher CRP level is associated with older age and greater BMI (25, 54). However, after co-varying for age and BMI, NLR remained significantly greater in patients with elevated relative to patients with normal CRP levels suggesting that CRP is a marker of brain illness/dysfunction in these cases and not simply a reflection of aging or excess weight. There was no significant difference between elevated and normal CRP groups in relation to antipsychotic dosage, supporting meta-analytical findings that CRP is not strongly modulated by antipsychotic dose (25). In contrast to previous studies that have shown smoking and drug use to be related to elevated CRP (55, 56), we found no significant difference between the patients with acute psychosis who displayed elevated and normal CRP in the proportion of smokers, drug users or poly substance abusers. Thus, the potential confounds did not appear to drive our findings in relation to the effects of CRP in this sample of acutely ill psychosis patients.

Other limitations include the lack of symptom severity measures, cognitive assessments and a restricted number of inflammation markers assessed. However, a common difficulty faced when working with acutely psychotic individuals admitted to inpatient hospitals is that these patients are frequently too unwell to complete standardized assessments. These patients met the clinical criteria for admission to the Mental Health Intensive Care Unit at intake, which is indicative of severe symptomatology and dysfunction.

In sum, after covarying for age and BMI, elevated CRP, and NLR appear to be characteristic of a substantial subset of people with acute psychosis. The findings from Study 1 support the hypothesis that a large subset (~60%) of individuals with acute psychosis display elevated markers of inflammation which is sustained across admissions for acute psychosis. Next, in Study 2 (below) we tested the relationship of CRP to symptoms, cognition and brain cortical thickness in a chronically ill sample of people with schizophrenia.

## Study 2: CRP in chronically ill patients with schizophrenia

### Materials and methods

#### Study design

##### Participants

The second study is derived from baseline data collected in our previously published clinical trial of a hormonal based treatment for schizophrenia (57). The current work represents our first assessment of CRP as an assay of immune system function in schizophrenia and the degree to which cognitive, symptom, daily function, and brain volume variables differ in relation to CRP in this sample. Only participants who provided a sufficient quantity of blood for analysis of CRP were included.

##### Chronically ill patients with schizophrenia

Eighty-five adult outpatients (51 males, 34 females) with schizophrenia or schizoaffective disorder, between 20 and 51 years of age, were included. All participants had been receiving antipsychotics for at least 1 year before entering the study (see Table 6 for the numbers of people receiving each antipsychotic or combination of antipsychotics). A diagnosis of schizophrenia (*n* = 54) or schizoaffective disorder (*n* = 31) was determined using the Structured Clinical Interview for Diagnostic and Statistical Manual IV-TR Axis I Disorders (SCID) (58) by a clinician trained in administration of the SCID and was confirmed independently by another clinician. Patients with a concurrent Axis I psychiatric diagnosis, a history of substance abuse or dependence (within the past 5 years), head injuries with loss of consciousness, seizures, central nervous system infection, untreated diabetes, or hypertension, or mental retardation were excluded. Women were excluded if they were pregnant. Seventy-four patients had their height and weight collected for BMI calculation. Sixty-nine patients also provided information about whether they were a smoker or non-smoker.

**Table 6:** Antipsychotic medication breakdown in the chronically ill patients with schizophrenia.

	***n***
Clozapine	19
Olanzapine	8
Clozapine/amisulpride	6
Risperidone	5
Amisulpride	4
Paliperidone depot	4
Aripiprazole	3
Clozapine/aripiprazole	3
Ziprasidone	3
Zuclopenthixol depot	3
Clozapine/paliperidone	2
Clozapine/risperidone depot	2
Quetiapine/paliperidone	2
Risperidone/quetiapine	2
Risperidone depot/risperidone	2
Risperidone depot	2
Zuclopenthixol depot/quetiapine	2
Amisulpride/quetiapine	1
Asenapine/aripiprazole	1
Clozapine/chlorpromazine	1
Clozapine/haloperidol	1
Flupenthixol	1
Haloperidol	1
Olanzapine/paliperidone	1
Paliperidone	1
Quetiapine	1
Risperidone depot/olanzapine	1
Risperidone depot/amisulpride	1
Zuclopenthixol/quetiapine	1
Zuclopenthixol depot/olanzapine	1

##### Healthy comparison group

Seventy-one healthy adults (34 males, 37 females) between 20 and 50 years of age were included. Exclusion criteria included a personal history of or a first-degree relative with a DSM-IV Axis I psychiatric diagnosis, history of substance abuse or dependence (within the past 5 years), head injuries with loss of consciousness, seizures, central nervous system infection, mental retardation, or untreated diabetes or hypertension.

#### Assessments

##### Cognitive assessments

All participants were administered a four subtest version of the Wechsler Adult Intelligence Scale – Third Edition (WAIS-III) (59) (consisting of Arithmetic, Similarities, Picture Completion and Digit Symbol subtests) as an estimate of current IQ, and the Wechsler Test of Adult Reading (WTAR) (60) as an estimate of premorbid IQ in schizophrenia. Working memory, verbal concept formation, visual perceptual organization, verbal fluency, attention/perceptual-motor processing speed and immediate and delayed verbal memory were assessed using WAIS-III Letter Number Sequencing (LNS) and Arithmetic, WAIS-III Similarities, WAIS-III Picture Completion, the Controlled Oral Word Association Test (COWAT) letter fluency (61), Form A of the Trail Making Test (TMT-A) (62) and Logical Memory I and II (LMI and LMII) of the Wechsler Memory Scale—Revised (63), respectively. TMT-A was incorporated into the battery after initiation of the study, which resulted in a lower total number of patients (*n* = 64) completing this measure.

##### Symptom and functional assessments

Symptom severity was assessed in patients using the Positive and Negative Syndrome Scale (PANSS) (64). Inter-rater reliability for the PANSS was high with an average intra-class correlation coefficient of 0.90. Negative emotional states, daily function and quality of life were assessed with the 21-item version of the Depression, Anxiety and Stress Scale (DASS-21) (65), the Short Form 36 Version 2 Health Survey Questionnaire (SF-36v2) (66), and the Schizophrenia Quality of Life Scale (SQLS) (67), respectively.

##### Blood collection and processing

Peripheral venous blood was collected from all participants in 8 ml Serum-separating tubes (SST Vacutainer, Becton Dickinson, Franklin Lakes, NJ) and 9 ml ethylenediaminetetraacetic acid (EDTA) tubes (Vacuette Vacutainer, Greiner Bio-One, Kremsmünster, Austria). Plasma was collected from EDTA tubes via centrifugation for 15 min at 2000 x g. SST tubes were incubated at room temperature for 30 min to allow for the blood to clot. Upon clotting, the serum was then collected via centrifugation for 5 min at 2000 x g. Plasma and serum were then aliquoted into protein low-binding tubes (Eppendorf, Hamburg, Germany) and stored at −80°C until the day of the assay.

CRP was measured in plasma using a high-sensitivity Enzyme-linked immunosorbent assay (ELISA) according to the manufacturer's instructions (IBL-international, Hamburg, Germany). Ten microliters of plasma was diluted serially up to 1:1,000 and each assay was run back to back with samples in duplicate by the same investigator, who was blind to the diagnosis. A five-point standard curve was generated using 10, 5, 1, 0.4, and 0 μg/ml calibrators that were prepared by the manufacturer. The average coefficient variance across all plates was 3.23%. The sample reads ranged from 0.06 mg/L to 23.92 mg/L. A minimum detectable value replacement of 0.003 was used for 13 data points that were below the respective minimal detectable value on all protein analytes.

##### MRI data collection and processing

From the total number of participants, 32 patients and 19 controls did not receive an MRI scan for reasons including electing not to receive an MRI scan, not able to physically fit in the MRI scanner, and/or experiencing claustrophobia. Thus, a subsample of participants (44 patients, 51 healthy controls) received a structural MRI scan of the brain. The MRI scans were performed using a 3-Tesla Phillips Achieva scanner with an 8-channel head coil located at Neuroscience Research Australia (Randwick, NSW). T1 weighted high-resolution anatomical scans were obtained (1 mm slice thickness, no gap, 180 slices, TR = 5.4 ms, TE = 2.4 ms, field of view 256 mm). After visual inspection of scans for motion and other artifacts and neuroanatomical abnormalities, data from 41 patients and 51 controls were analyzed for cortical thickness. The scans were processed using Mac OSX 10.8 running FreeSurfer software (version 5.1.0, Athinoula A. Martinos Center for Biomedical Imaging, Massachusetts, USA). A detailed methodological description of the FreeSurfer cortical reconstruction, volumetric segmentation and subsequent deformable procedures (including published references) is available at *http://surfer.nmr.mgh.harvard.edu/*. Processed scans were visually evaluated and manually edited as necessary by trained personnel. Cortical thickness data for each region were obtained based on the FreeSurfer Desikan-Killiany atlas.

#### Statistical analyses

Data analysis was performed using SPSS version 23. Where possible, age- and sex-adjusted scaled scores were used for the cognitive tests. Extreme outliers (≥ ± 2 standard deviations from the mean) for the CRP variable were excluded from the analysis (total excluded *n* = 10, schizophrenia group *n* = 9, healthy control group *n* = 1).

Demographic, clinical, cognitive, symptom severity, negative emotional states, quality of life, and daily function scores were compared between the chronically ill schizophrenia group and healthy control group using *t*-tests or χ^2^ as appropriate.

Based on clinical cut-off scores, all participants were grouped into two categories: “normal CRP” with CRP < 3 mg/L and “elevated CRP” with CRP ≥ 3 mg/L (36). χ^2^ tests were performed to determine if there were significant differences in the numbers of participants among the CRP groups in patients and controls. An ANOVA was performed between the acute psychosis patient group (from Study 1) and the chronically ill schizophrenia patient group (from Study 2) to determine the extent to which CRP levels differed between these patient groups. Demographic, clinical, cognitive, symptom severity, negative emotional states, quality of life, and daily function scores were compared among normal CRP groups in healthy controls and normal and elevated CRP groups in schizophrenia patients using univariate ANOVAs with False Discovery Rate (FDR) corrections for multiple comparisons (68). Effect size calculations were reported as Cohen's *d* (50).

Further analyses included paired *t*-tests in a subset of 46 patients with schizophrenia who had elevated CRP (*n* = 23) and normal CRP (*n* = 23) who were matched within 2 points on BMI to compare performance on any measures that were significantly different between the elevated and normal CRP groups (i.e., WAIS-III IQ and Arithmetic) to control for BMI.

FreeSurfer cortical thickness estimates for the left and right hemispheres were summed to create a composite measure for each brain region. Simple linear regression models (backward elimination) were used to model potential predictors of cortical thickness. Predictor variables consisted of CRP, diagnostic group, age at sampling and sex. The criterion for backward elimination was *F* ≥ 0.1.

#### Study approval

All participants provided written informed consent consistent with the Declaration of Helsinki which was obtained after the nature and possible consequences of the study were explained. The study was conducted under protocols approved by the University of New South Wales (07/121 and 09/187), South Eastern Sydney Local Health District (07-259) Human Research Ethics Committees and the Queen Elizabeth Hospital Ethics and Human Research Committee, Adelaide (2010188).

### Results

#### Sample characteristics

The number of chronically ill patients with schizophrenia receiving different antipsychotics are provided in Table 6. Cohort demographics, cognitive, symptom, negative emotional states, and daily functioning scores, and CRP levels are provided in Table 7. Relative to healthy controls, patients with schizophrenia were significantly older (although the groups were only 9.4% different in age) and had significantly less education. Patients with schizophrenia also had significantly lower (worse) scores for cognitive, negative emotions, and daily functioning relative to controls. As predicted, the patients with schizophrenia had significantly elevated CRP levels relative to controls (an 88.9% increase, see Table 7). There were no significant differences between schizophrenia and control groups in relation to sex or ethnicity ratios. The patients displayed mild to moderate symptom severity based on Positive and Negative Syndrome Scale (PANSS) scores, were chronically ill with a typical mean age of onset and the majority of patients were receiving second generation antipsychotic medication (see Table 6).

**Table 7:** Demographics, cognitive, symptom severity, negative emotional states, daily function measures, and C-reactive protein levels in chronically ill patients with schizophrenia and healthy controls.

	***n***	**Schizophrenia**	***n***	**Healthy controls**	***t*-test or χ^2^**	***p***	**Cohen's d**
Age (years)	85	35.8 (8.6)	71	32.2 (8.3)	*t* = 2.68	< 0.01^*^	0.43
**Sex (number)**	85		71				
Male/Female		51/34		34/37	χ^2^ = 2.29	0.13	-
**Ethnicity (number)**	85		71				
Caucasian/Other		74/11		59/12	χ^2^ = 0.83	0.51	-
Education (years)	85	12.5 (2.3)	71	14.7 (2.3)	*t* = 5.72	< 0.01^*^	0.96
Age of illness onset (years)	85	22.8 (5.8)	–	–	–	–	–
Illness duration (years)	85	13.0 (7.5)	–	–	–	–	–
CPZ equivalent (mg)	85	563.2 (484.2)	–	–	–	–	–
**IMMUNE FUNCTIONING**							
CRP	76	3.4 (3.2)	70	1.8 (2.7)	*t* = 3.29	0.01^*^	0.55
**COGNITIVE TESTS**							
WTAR	85	101.9 (9.2)	71	107.7 (9.0)	*t* = 3.96	< 0.01^*^	0.64
WAIS-III IQ	85	91.1 (12.8)	71	107.3 (15.4)	*t* = 7.20	< 0.01^*^	1.15
WAIS-III Similarities	85	9.3 (2.9)	71	10.8 (2.7)	*t* = 3.38	< 0.01^*^	0.54
WAIS-III Arithmetic	85	7.9 (3.2)	71	10.9 (3.2)	*t* = 5.67	< 0.01^*^	0.94
WAIS-III Digit Symbol	85	7.0 (2.4)	71	11.5 (3.4)	*t* = 9.49	< 0.01^*^	1.53
WAIS-III Picture Completion	85	8.6 (2.4)	71	10.8 (2.8)	*t* = 5.22	< 0.01^*^	0.84
WAIS-III LNS	85	8.1 (2.7)	71	11.1 (2.8)	*t* = 6.82	< 0.01^*^	1.09
WMS-R LMI	85	7.9 (3.5)	71	11.9 (3.3)	*t* = 7.24	< 0.01^*^	1.18
WMS-R LMII	85	6.1 (3.3)	71	10.4 (3.5)	*t* = 7.71	< 0.01^*^	1.26
TMT-A	64	39.0 (13.2)	56	25.9 (8.9)	*t* = −6.46	< 0.01^*^	1.16
COWAT verbal fluency	85	37.3 (11.0)	71	41.6 (11.4)	*t* = 2.40	0.02^*^	0.38
**SYMPTOM AND DAILY FUNCTION**							
**PANSS**							
Positive	85	15.2 (4.5)	–	–	–	–	–
Negative	85	14.1 (5.9)	–	–	–	–	–
General	85	30.3 (8.5)	–	–	–	–	–
Total	85	59.5 (15.9)	–	–	–	–	–
**DASS**							
Depression	85	12.6 (10.0)	66	3.2 (5.1)	*t* = 7.52	< 0.01^*^	1.18
Anxiety	85	10.5 (8.0)	66	2.6 (3.9)	*t* = 7.95	< 0.01^*^	1.26
Stress	85	14.9 (9.9)	66	6.4 (7.1)	*t* = 6.16	< 0.01^*^	0.99
SF-36v2 total	85	113.2 (23.8)	66	141.4 (10.1)	*t* = 9.78	< 0.01^*^	1.54
SQLS total	85	47.6 (18.5)	66	22.4 (10.3)	*t* = 10.44	< 0.01^*^	1.68

*CPZ, chlorpromazine; WTAR, Wechsler Test of Adult Reading; WAIS-III, Wechsler Adult Intelligence Scale 3rd Edition; WMS-R LMI, Wechsler Memory Scale Revised Logical Memory I; WMS-R LMII, Wechsler Memory Scale - Revised Logical Memory II; LNS, Letter Number Sequencing; TMT-A, Trail Making Test Form A; COWAT, Controlled Oral Word Association Test; PANSS, Positive and Negative Syndrome Scale; DASS, Depression Anxiety and Stress Scale; SF-36v2, Short Form 36 Version 2 Health Survey Questionnaire; SQLS, Schizophrenia Quality of Life Scale; CRP, C-reactive protein. Unless otherwise specified, values refer to means and s.d. in parentheses*.

* *p < 0.05*.

#### CRP levels

There was a significant difference in the proportions of people with elevated CRP in schizophrenia and healthy control groups; $\chi_{\left(1\right)}^{2}$ = 9.16, *p* < 0.01. Forty three percent of people with schizophrenia had CRP ≥ 3 mg/L (*n* = 33; CRP range 3.0–12.3) compared to 20% of healthy controls with CRP ≥ 3 mg/L (*n* = 14; CRP range 3.0–13.5).

**Figure 1:**
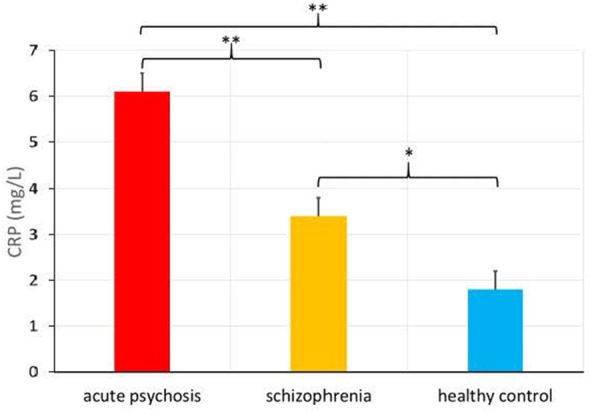
C-Reactive Protein (CRP) levels (in mg/L) in patients admitted for an acute psychotic episode, chronically ill patients with schizophrenia, and healthy controls (means provided with standard error), *p < 0.01, **p < 0.001.

There was also a significantly higher CRP level in the acute psychosis patient group from Study 1 (*M* = 6.1, *SD* = 6.7) relative to the chronically ill patient group from Study 2 [*M* = 3.4, *SD* = 3.2, *F*_(1, 248)_ = 11.36, *p* = 0.001, see Figure 1], as well as a significant difference in the proportions of people in the elevated versus normal CRP groups among acute psychosis patients, chronically ill patients, and healthy controls, $\chi_{\left(2\right)}^{2}$ = 11.2, *p* = 0.004.

#### Subgrouping based on CRP levels

As shown in Table 8, there were no significant differences between elevated and normal CRP groups in healthy controls in relation to any of the demographic, negative emotional states, daily function, quality of life, or cognitive measures after adjustment for multiple comparisons. Table 9 shows that after adjustment for multiple comparisons, there were significant differences between the elevated and normal CRP groups in patients with schizophrenia in relation to sex, BMI, current IQ (WAIS-III IQ) and working memory (i.e., WAIS-III Arithmetic subtest), with a large effect size for working memory such that performance was significantly poorer in the elevated CRP group of patients. Paired *t*-tests revealed a significant difference in working memory (WAIS-III Arithmetic) remained between the patients with schizophrenia displaying normal and elevated CRP who were matched on BMI (see Table 10). There was no significant difference between the matched groups on current IQ (WAIS-III IQ).

**Table 8:** Comparison of demographic, cognitive, negative emotional states, daily function, and quality of life measures between normal and elevated CRP groups in healthy controls.

	***n***	**“Normal” CRP < 3mg/L**	***n***	**“Elevated” CRP ≥3mg/L**	***F* or χ^2^**	***p***	**FDR-adjusted p**	**Cohen's d**
**CLINICAL VARIABLES**								
Age (years)	56	32.3 (8.2)	14	32.1 (9.1)	*F* = 0.00	0.96	0.96	0.02
**Sex (number)**								
Male/Female		27/29		6/8	χ^2^ = 0.13	0.72	0.96	-
Ethnicity (number)					χ^2^ = 1.23	0.27	0.96	*-*
Caucasian/Other		45/11		13/1				
Education (years)	56	14.7 (2.1)	14	14.4 (3.0)	*F* = 0.20	0.66	0.96	0.12
**READING AND CURRENT IQ**								
WTAR	56	109.3 (8.3)	14	103.1 (8.8)	*F* = 6.13	0.02	0.13	0.76
WAIS-III IQ	56	109.3 (13.8)	14	100.8 (20.0)	*F* = 3.53	0.07	0.13	0.49
**COGNITIVE TESTS**								
WAIS-III Similarities	56	11.2 (2.7)	14	9.6 (2.3)	*F* = 4.21	0.04	0.13	0.64
WAIS-III Arithmetic	56	11.1 (3.0)	14	10.1 (3.6)	*F* = 1.08	0.30	0.36	0.30
WAIS-III Digit Symbol	56	11.8 (3.2)	14	10.0 (3.6)	*F* = 3.47	0.07	0.13	0.53
WAIS-III Picture Completion	56	11.1 (2.6)	14	9.5 (3.5)	*F* = 3.71	0.06	0.13	0.52
WAIS-III LNS	56	11.2 (3.0)	14	10.4 (1.9)	*F* = 0.98	0.33	0.36	0.32
WMS-R LMI	56	12.3 (3.3)	14	10.7 (3.2)	*F* = 2.62	0.11	0.17	0.49
WMS-R LMII	56	10.9 (3.5)	14	8.6 (2.9)	*F* = 5.22	0.03	0.13	0.72
TMT-A	42	24.8 (9.2)	13	28.4 (6.7)	*F* = 1.74	0.19	0.26	0.45
COWAT verbal fluency	56	42.4 (11.4)	14	39.4 (10.7)	*F* = 0.83	0.37	0.37	0.27
**NEGATIVE EMOTION AND DAILY FUNCTION**								
**DASS**								
Depression	51	3.0 (5.1)	14	4.1 (5.5)	*F* = 0.55	0.46	0.63	0.21
Anxiety	51	2.5 (3.8)	14	4.1 (5.5)	*F* = 0.47	0.50	0.63	0.34
Stress	51	6.1 (7.2)	14	8.0 (6.9)	*F* = 0.80	0.38	0.63	0.27
SF-36 v2 Total	51	142.4 (8.1)	14	136.6 (14.4)	*F* = 3.92	0.05	0.25	0.50
SQLS Total	51	22.4 (10.5)	14	23.4 (10.0)	*F* = 0.10	0.75	0.75	0.10

*WTAR, Wechsler Test of Adult Reading; WAIS-III, Wechsler Adult Intelligence Scale 3rd Edition; LNS, Letter Number Sequencing; WMS-R LMI, Wechsler Memory Scale Revised Logical Memory I; WMS-R LMII, Wechsler Memory Scale - Revised Logical Memory II; TMT-A, Trail Making Test Form A; COWAT, Controlled Oral Word Association Test; DASS, Depression Anxiety and Stress Scale; SF-36v2, Short Form 36 Version 2 Health Survey Questionnaire; SQLS, Schizophrenia Quality of Life Scale*.

**Table 9:** Comparison of demographic, clinical, cognitive, symptom severity, negative emotional states, daily function and quality of life measures between normal and elevated CRP groups in patients with schizophrenia.

	***n***	**“Normal” CRP < 3 mg/L**	***n***	**“Elevated” CRP ≥3 mg/L**	***F* or χ^2^**	***p***	**FDR-adjusted p**	**Cohen's d**
**CLINICAL VARIABLES**								
Age (years)	43	36.3 (8.8)	33	34.9 (8.0)	*F* = 0.49	0.49	0.69	0.17
**Sex (number)**								
Male/Female		32/11		14/19	χ^2^ = 8.00	0.005	0.03^*^	-
**Ethnicity (number)**								
Caucasian/Other		35/8		30/3	χ^2^ = 1.37	0.24	0.60	-
Education (years)	43	13.0 (2.4)	33	12.3 (2.3)	*F* = 1.73	0.19	0.60	0.30
Smokers (number)		18		14	χ^2^ = 0.82	0.84	0.84	-
CPZ equivalent	43	486.9 (435.6)	33	562.7 (461.8)	*F* = 0.54	0.47	0.69	0.17
Illness duration	43	12.7 (8.0)	33	13.4 (7.2)	*F* = 0.16	0.69	0.77	0.09
Age of onset	43	23.3 (6.1)	33	22.1 (5.1)	*F* = 0.89	0.35	0.69	0.22
BMI	38	29.3 (6.2)	28	33.7 (6.0)	*F* = 8.22	0.006	0.03^*^	0.72
**IMMUNE FUNCTIONING**								
NLR	42	2.4 (1.2)	31	2.5 (1.0)	*F* = 0.36	0.55	0.69	0.09
**PREMORBID AND CURRENT IQ**								
WTAR	43	102.7 (10.4)	33	102.2 (8.0)	*F* = 0.40	0.84	0.84	0.05
WAIS-III IQ	43	95.1 (12.5)	33	87.2 (11.7)	*F* = 7.87	0.006	0.03^*^	0.65
**COGNITIVE TESTS**								
WAIS-III Similarities	43	9.8 (2.8)	33	9.0 (3.1)	*F* = 1.22	0.27	0.41	0.25
WAIS-III Arithmetic	43	9.1 (3.1)	33	6.6 (3.0)	*F* = 11.57	0.001	0.01^*^	0.79
WAIS-III Digit Symbol	43	7.7 (2.4)	33	6.4 (2.2)	*F* = 5.97	0.02	0.07	0.57
WAIS-III Picture Completion	43	8.8 (2.4)	33	8.3 (2.2)	*F* = 1.04	0.31	0.41	0.22
WAIS-III LNS	43	8.5 (2.7)	33	7.5 (2.8)	*F* = 2.74	0.10	0.28	0.38
WMS-R LMI	43	8.1 (3.4)	33	7.4 (3.4)	*F* = 0.98	0.32	0.41	0.24
WMS-R LMII	43	6.3 (3.2)	33	5.6 (3.1)	*F* = 0.91	0.34	0.41	0.22
TMT-A	33	38.0 (12.1)	28	41.1 (12.6)	*F* = 0.83	0.37	0.41	0.25
COWAT verbal fluency	43	39.2 (11.1)	33	36.3 (11.1)	*F* = 1.28	0.26	0.41	0.26
**SYMPTOMS/DAILY FUNCTION**								
**PANSS**								
Positive	43	14.7 (4.1)	33	15.6 (4.7)	*F* = 0.72	0.40	0.72	0.20
Negative	43	13.9 (5.7)	33	14.6 (6.6)	*F* = 0.26	0.62	0.77	0.16
General	43	29.7 (8.6)	33	31.5 (8.5)	*F* = 0.79	0.38	0.72	0.21
Total	43	58.3 (15.4)	33	61.6 (16.7)	*F* = 0.81	0.37	0.72	0.21
**DASS**								
Depression	43	13.5 (9.5)	33	12.1 (10.7)	*F* = 0.40	0.53	0.77	0.15
Anxiety	43	10.1 (7.7)	33	12.1 (8.2)	*F* = 1.22	0.27	0.72	0.26
Stress	43	15.4 (9.6)	33	15.2 (9.9)	*F* = 0.01	0.95	0.95	0.01
SF-36 v2 Total	43	115.9 (18.7)	33	107.9 (29.1)	*F* = 2.07	0.16	0.72	0.33
SQLS Total	43	48.7 (16.6)	33	47.0 (19.3)	*F* = 0.17	0.68	0.77	0.10

*CPZ, chlorpromazine; BMI, body mass index; NLR, neutrophil-to-lymphocyte ratio; WTAR, Wechsler Test of Adult Reading; WAIS-III, Wechsler Adult Intelligence Scale 3rd Edition; LNS, Letter Number Sequencing; WMS-R LMI, Wechsler Memory Scale Revised Logical Memory I; WMS-R LMII, Wechsler Memory Scale - Revised Logical Memory II; TMT-A, Trail Making Test Form A; COWAT, Controlled Oral Word Association Test; PANSS, Positive and Negative Syndrome Scale; DASS, Depression Anxiety and Stress Scale; SF-36v2, Short Form 36 Version 2 Health Survey Questionnaire; SQLS, Schizophrenia Quality of Life Scale*.

* *p < 0.05*.

**Table 10:** Comparison of current IQ and working memory measures between normal and elevated CRP schizophrenia groups matched on BMI.

	***n***	**“Normal” CRP < 3mg/L**	***n***	**“Elevated” CRP ≥3mg/L**	***df***	***t***	***p***
BMI	23	32.2 (5.8)	23	32.3 (5.8)	22	−0.55	0.59
WAIS-III IQ	23	95.8 (11.6)	23	89.4 (11.3)	22	1.64	0.12
Arithmetic	23	9.2 (2.8)	23	7.0 (3.0)	22	2.69	0.01^*^

*WAIS-III IQ, Wechsler Adult Intelligence Scale, 3rd Edition*.

* *p = 0.01*.

#### Cortical thickness analysis

**Figure 2:**
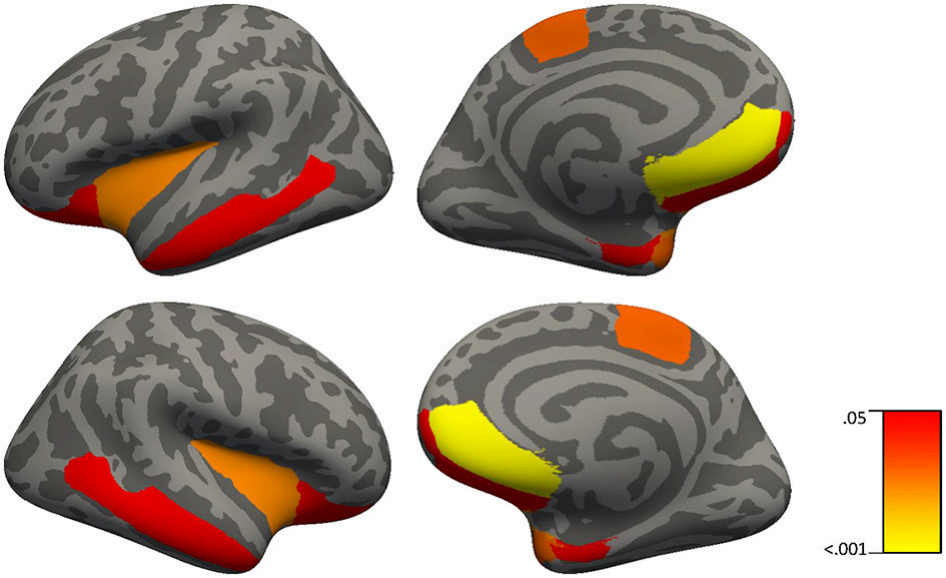
Brain regions in which C-reactive protein significantly predict cortical thickness in 51 healthy controls and 41 patients with schizophrenia using simple linear regressions with backward elimination (regions highlighted represent significant effects at FDR *p* < 0.04).

Results from the regression analysis using diagnostic group, age, sex, and CRP revealed significant contributions of the independent variables to the prediction of cortical thickness in several frontal and temporal regions in addition to the insula and paracentral gyrus (see Table 11 and Figure 2). In relation to our main variable of interest, CRP significantly predicted cortical thickness in most (8/9) regions, with CRP predicting from 3.9% of the variance in the frontal pole and middle temporal lobe to 7.7% of the variance in the temporal pole. The relationships between CRP levels and cortical thickness were inverse, demonstrating that as CRP levels increased, cortical thickness decreased.

**Table 11:** Results of regression analysis showing significant contributions of diagnostic (Dx) group (schizophrenia or control), age, sex, and C-Reactive Protein (CRP) to cortical thickness.

**Lobe**	**Brain Region**	**Model**		**Coefficients**				
		***R***	**Adjusted R^2^ (SE)**	**Predictors**	**% Variance**	**Std Beta Coef**	***p***	**FDR**
Frontal	Frontal pole	0.378	0.115 (0.485)	CRP	3.9	−0.199	0.044	0.045
				Age	7.4	−0.275	0.006	0.03
	Medial orbital frontal	0.538	0.274 (0.319)	CRP	7.5	−0.275	0.002	0.016
				Age	18.8	−0.435	< 0.001	0.005
	Lateral orbitofrontal	0.558	0.288 (0.294)	CRP	4.1	−0.219	0.021	0.039
				Age	9.6	−0.328	0.001	0.015
				Diagnosis	4.3	−0.234	0.019	0.036
	Superior frontal	0.601	0.34 (0.286)	CRP	2.5	−0.170	0.062	0.047
				Age	12.3	−0.370	< 0.001	0.012
				Diagnosis	6.3	−0.283	0.004	0.023
Temporal	Temporal pole	0.392	0.135 (0.436)	CRP	7.7	−0.279	0.005	0.028
				Age	6.1	−0.248	0.012	0.033
	Middle temporal	0.610	0.351 (0.285)	CRP	3.9	−0.212	0.019	0.037
				Age	11.2	−0.353	< 0.001	0.006
				Diagnosis	6.3	−0.282	0.003	0.021
	Entorhinal	0.414	0.153 (0.608)	CRP	4.6	−0.231	0.026	0.04
				Diagnosis	6.3	−0.270	0.009	0.031
Other	Insula	0.666	0.425 (0.269)	CRP	5.5	−0.251	0.004	0.024
				Age	16.0	−0.419	< 0.001	0.014
				Diagnosis	5.2	−0.258	0.004	0.024
	Paracentral	0.419	0.158 (0.324)	CRP	7.4	−0.274	0.005	0.026
				Age	8.4	−0.291	0.005	0.026

*Only those brain regions showing significant effects are reported in the table*.

As would be expected, age was generally the strongest predictor of cortical thickness in most regions showing significant results, predicting from 6.1% of the variance in the temporal pole to 18.8% of the variance in the medial orbital frontal cortex. Also, as expected, diagnostic group predicted from 4.3% of cortical thickness in the lateral orbital frontal cortex to 6.3% of cortical thickness in superior frontal gyrus, middle temporal lobe, and entorhinal cortex.

### Discussion

In relation to elevated CRP, there was a significant difference between the proportions of chronically ill patients with schizophrenia (43%) and healthy controls (20%) who could be classified as having elevated levels of inflammation. These findings suggest the existence of a low-grade systemic inflammation in nearly half of the patients studied. This is consistent with our report of a similar proportion (~40%) of chronically ill patients displaying elevated cytokine mRNA expression in post-mortem prefrontal cortex tissue (17) and displaying elevated cytokine mRNA expression in blood (69). On average, the chronically ill patients with schizophrenia displayed significantly lower CRP levels than the acute psychosis group, supporting previous work showing that CRP may decrease following antipsychotic treatment and resolution of acute psychotic episodes (42). Thus, a substantial proportion of chronically ill patients with schizophrenia display elevated peripheral inflammation markers and CRP in particular.

Some studies have shown that elevated CRP is associated with poorer cognitive performance (verbal memory, global cognition) both in patients with schizophrenia (40) and healthy older adults (70). In the current study, the elevated CRP schizophrenia group displayed significantly worse performance on a measure of working memory compared to the normal CRP schizophrenia group after matching for BMI. These results suggest that immune-related mechanisms may be a contributing factor to working memory/reasoning deficits (which are known to be particularly dependent on a healthy prefrontal cortex) in schizophrenia. Given that working memory deficits are particularly predictive of long-term functional outcome (71), the influence of elevated CRP on working memory/reasoning suggests that adjunctive anti-inflammatory treatments may reduce cognitive deficits and improve daily function in a substantial subset of patients with schizophrenia. Our results suggest that measuring CRP levels in blood of chronically ill people with schizophrenia would also be an indicator of impaired prefrontal cortex function.

Contrary to our hypotheses, there were no significant differences between elevated and normal CRP patient groups in relation to symptom severity as measured by PANSS ratings. This finding is discordant with results from a previous study which reported greater severity of negative and general symptoms in patients with schizophrenia displaying elevated CRP levels (42). However, this difference may be due to inclusion/exclusion criteria and sampling differences. For instance, the sample used in the previous study consisted of patients with moderately high levels of symptom severity (42), whereas, the patients in the current sample displayed mild to moderate levels of symptom severity. In support of this view, the results of the current study are consistent with other studies that have failed to find an association between inflammation markers and symptom severity in patients with mild to moderate symptom severity (40). Thus, the presence of greater illness severity appears to be necessary to reveal the relationship between illness severity and inflammation.

Our study is the first to assess brain cortical thickness measures in relation to CRP levels in patients with schizophrenia and healthy controls. CRP levels made a significant contribution to cortical thickness in 8 out of 9 regions including the frontal pole, medial orbital frontal, lateral orbital frontal, middle temporal cortices, temporal pole, entorhinal cortex, insula, and paracentral gyrus in both patients and controls. An inverse relationship between CRP levels and cortical thickness in prefrontal regions is consistent with the elevated CRP patients with schizophrenia displaying a significantly worse working memory performance relative to patients with schizophrenia who display normal CRP levels; however, it is typically aberrant activity in the dorsolateral prefrontal cortex, not other prefrontal regions that is associated with changes in working memory in people with schizophrenia (72). However, others have shown activation of the inferior frontal gyrus in healthy adults and decreased blood flow to the middle temporal gyrus in coronary artery disease during mental stress induced by arithmetic (73) suggesting involvement of both ventral regions and temporal regions to math challenges. Since thinner cortex in temporal regions also showed a relationship to elevated CRP, CRP might have been expected to correlate with episodic memory performance. Although there was such a relationship (see Table 8 for delayed recall in controls) it did not survive correction for multiple comparisons suggesting that this relationship between CRP and delayed memory was less robust than that for working memory and prefrontal cortex. One limitation of this study would be in relation to the restricted number of inflammation markers assessed in our study; however, we have reported on the relationship of other peripheral inflammation markers (e.g., IL-1β) to cognition and brain volume in a smaller sample of chronically ill patients with schizophrenia (30). Overall, these results in relation to CRP levels and cortical thickness provide further support for the role of inflammatory processes especially influencing prefrontal brain structure and cognition in patients with schizophrenia.

## General discussion

We assessed the proportions of patients displaying a marker of inflammation (CRP) in two independent samples (in chronically ill patients with schizophrenia and over repeated admissions in patients experiencing an acute psychotic episode) and the ability of CRP to predict cortical thickness. The results of our study in patients admitted for an acute psychotic episode showed that acute psychosis is often associated with a relatively high level of systemic inflammation as measured by elevated CRP, NLR and positive ANA titers. In our study of chronically ill patients with schizophrenia, we showed that CRP was also significantly elevated relative to healthy controls. A larger proportion of individuals (~60%) with acute psychosis had elevated levels of CRP, compared to ~44% of the chronically ill patients with schizophrenia. This is consistent with findings that elevated CRP may decrease with resolution of psychotic symptoms (45). Some healthy controls also displayed elevated CRP, although the proportion (20%) was significantly lower than the proportions displaying elevated CRP in chronically ill patients with schizophrenia and those patients admitted for an acute psychotic episode. The finding of elevated CRP in some healthy controls is not unexpected as slightly increased and/or chronic immune activation can be found in otherwise healthy samples given that common inflammatory processes, including allergies, asthma, or arthritis may be present in otherwise healthy individuals, e.g., the prevalence of arthritis is 22.7% annually in the US (74).

The chronically ill patients with schizophrenia who displayed elevated CRP had significantly worse working memory performance. This is consistent with other studies indicating an association between CRP and cognitive deficits (such as global cognition, attention/psychomotor speed, language/executive function, memory, and visual spatial ability) in other populations including individuals with cardiovascular disease (75), obstructive sleep apnoea (76), and mild cognitive impairment (77). Given that BMI also differed between elevated and normal CRP patient groups in our study, we showed that after matching on BMI the significant difference in arithmetic performance remained between the elevated and normal CRP patients with schizophrenia; suggesting that factors intrinsic to schizophrenia and inflammation may underlie the cognitive deficits in this subgroup of patients. The presence of a significant difference in cognitive variables between elevated and normal CRP groups in schizophrenia coupled with the lack of significant cognitive differences in the healthy sample also supports the hypothesis suggesting a role of inflammatory processes in schizophrenia.

In conclusion, the current results show that CRP levels are substantially elevated in acute psychosis involving the majority of patients who presented to a mental health intensive care inpatient ward. While we did find some reduction of inflammation across admissions, we saw continued significantly increased CRP in these readmitted patients and a lower, but still significant CRP elevation in chronically ill patients with schizophrenia. NLR remained significantly elevated in the elevated CRP group of patients admitted for an acute psychotic episode after covarying for BMI, suggesting that the elevated inflammation would not be fully explained by BMI in these acutely ill patients. Similarly, significant working memory differences remained between elevated and normal CRP chronically ill patient groups after covarying for BMI, again suggesting that cognitive deficits associated with inflammation were not due to elevated BMI. Importantly, we provide further novel evidence that elevated CRP is not occurring in isolation of brain changes. We found significant relationships among peripheral CRP and measures of human brain structure and function especially involving the frontal lobe suggesting that this highly evolved human brain region may be especially sensitive to inflammation. This current work supports our earlier work (30) on the relationship between worse verbal fluency, lower Broca's area volume and higher IL-1β levels in patients with schizophrenia, suggesting that anti-inflammatory drugs may be useful to include as adjunctive treatments in those patients showing elevated inflammatory markers (15, 78).

## Author contributions

IJ, CS, RV, HP, MO, RL, CG, CW, and TW designed and conducted the project. IJ and RB entered data, managed the database, analyzed and interpreted the data. RV and HP assisted with data entry and database management. RL, JB, CG, DL, CW, and TW assisted with interpretation of data. IJ wrote the initial draft of the manuscript, and all authors contributed comments and edited the manuscript.

### Conflict of interest statement

The authors declare that the research was conducted in the absence of any commercial or financial relationships that could be construed as a potential conflict of interest.
